# RETRACTION: Effect of Honey Dressing in the Management of Diabetic Foot Ulcers: A Meta‐Analysis

**DOI:** 10.1111/iwj.70224

**Published:** 2025-02-11

## Abstract

**RETRACTION**: LiS. , XiaoT. , YeN. , YangG. , ChenH. , LiangX. , LiT. , WangJ. , PengY. , LiY. , and LiuY. , “Effect of Honey Dressing in the Management of Diabetic Foot Ulcers: A Meta‐Analysis,” International Wound Journal 20, no. 7 (2023): 2626–2633, 10.1111/iwj.14135.36994798 PMC10410322

The above article, published online on 30 March 2023, in Wiley Online Library (http://onlinelibrary.wiley.com/), has been retracted by agreement between the journal Editor in Chief, Professor Keith Harding; and John Wiley & Sons Ltd. Following an investigation by the publisher, all parties have concluded that this article was accepted solely on the basis of a compromised peer review process. The editors have therefore decided to retract the article. The authors did not respond to our notice regarding the retraction.

**RETRACTION**: S. Li , T. Xiao , N. Ye , G. Yang , H. Chen , X. Liang , T. Li , J. Wang , Y. Peng , Y. Li , and Y. Liu , “Effect of Honey Dressing in the Management of Diabetic Foot Ulcers: A Meta‐Analysis,” International Wound Journal 20, no. 7 (2023): 2626–2633, 10.1111/iwj.14135.36994798 PMC10410322

The above article, published online on 30 March 2023, in Wiley Online Library (http://onlinelibrary.wiley.com/), has been retracted by agreement between the journal Editor in Chief, Professor Keith Harding; and John Wiley & Sons Ltd. Following an investigation by the publisher, all parties have concluded that this article was accepted solely on the basis of a compromised peer review process. The editors have therefore decided to retract the article. The authors did not respond to our notice regarding the retraction.

